# Development and characterization of anti-fibrotic natural compound similars with improved effectivity

**DOI:** 10.1007/s00395-022-00919-6

**Published:** 2022-03-02

**Authors:** Fabian Philipp Kreutzer, Anna Meinecke, Saskia Mitzka, Hannah Jill Hunkler, Lisa Hobuß, Naisam Abbas, Robert Geffers, Jan Weusthoff, Ke Xiao, Danny David Jonigk, Jan Fiedler, Thomas Thum

**Affiliations:** 1 Institute of Molecular and Translational Therapeutic Strategies (IMTTS), Center of Pharmacology and Toxicology, Hannover Medical School, Carl-Neuberg-Str.1, 30625 Hannover, Germany; 2 Fraunhofer Institute for Toxicology and Experimental Medicine (ITEM), Hannover, Germany; 3 Helmholtz Centre for Infection Research, Braunschweig, Germany; 4 Institute of Pathology, Hannover Medical School, Hannover, Germany; 5 Member of Biomedical Research in Endstage and Obstructive Lung Disease Hannover (BREATH), German Center for Lung Research, Hannover, Germany; 6 REBIRTH Center for Translational Regenerative Medicine, Hannover Medical School, Hannover, Germany

**Keywords:** Cardiac fibroblast, Cardiac fibrosis, Natural compounds, Bufalin, Lycorine, Heart failure, Homoharringtonine

## Abstract

Cardiac fibroblasts constitute the major cell type of the murine and human heart. Once activated, they contribute to an excessive deposition of extracellular matrix (ECM) leading to cardiac fibrosis and subsequently organ dysfunction. With the exception of the pulmonary drugs, nintedanib and pirfenidone, drugs specifically targeting anti-fibrotic pathways are scarce. We recently performed large library screenings of natural occurring compounds and identified first lead structures with anti-fibrotic properties in vitro and in vivo. In line, we now aimed to improve efficacy of these anti-fibrotic lead structures by combining in vitro validation studies and in silico prediction. Next to this combined approach, we performed large OMICs-multi-panel-based mechanistic studies. Applying human cardiac fibroblasts (HCF), we analysed 26 similars of the initially identified anti-fibrotic lead molecules bufalin and lycorine and determined anti-proliferative activity and potential toxicity in an array of in vitro and ex vivo studies. Of note, even at lower concentrations, certain similars were more effective at inhibiting HCF proliferation than nintedanib and pirfenidone. Additionally, selected similars showed low cytotoxicity on human iPS-derived cardiomyocytes and anti-fibrotic gene regulation in human ex vivo living myocardial slices. Further, array and RNA sequencing studies of coding and non-coding RNAs in treated HCFs revealed strong anti-fibrotic properties, especially with the lycorine similar lyco-s (also known as homoharringtonine), that led to a nearly complete shutdown of ECM production at concentrations 100-fold lower than the previously identified anti-fibrotic compound lycorine without inducing cellular toxicity. We thus identified a new natural compound similar with strong anti-fibrotic properties in human cardiac fibroblasts and human living heart tissue potentially opening new anti-fibrotic treatment strategies.

**Supplementary Information:**

The online version contains supplementary material available at 10.1007/s00395-022-00919-6.

## Introduction

In the last decades, cardiovascular diseases (CVD) became the leading cause of death worldwide with approx. 30% of all deaths attributed to CVD [53]. One major driver and representation of CVD is heart failure (HF), the inability of the heart to supply the body with the necessary amount of oxygenated blood, causing breathlessness, fatigue and swelling. An estimate of 64 million people worldwide are affected by HF, and morbidity as well as mortality remain high while quality of life remains poor [41, 49]. To date, therapeutic options for those HF patients with preserved ejection fraction (HFpEF) but enhanced stiffness of the heart are still absent [37].

A major underlying cause of HFpEF is cardiac fibrosis, marked by excessive deposition of extracellular matrix (ECM). Currently, there are two anti-fibrotic drugs, nintedanib and pirfenidone, which are approved for treatment of idiopathic pulmonary fibrosis (IPF). While both slow disease progression or prolong progression-free survival in IPF patients, adverse reactions limit the therapeutic potential [18, 40]. The potential therapeutic effect of pirfenidone in HF patients is currently under investigation, and a recent phase II clinical trial (NCT02932566) showed slight improvements of extracellular volume, a surrogate measurement of cardiac fibrosis, but not in hemodynamic parameters [28]. While this approach is promising, there is a serious and ever increasing need for novel therapeutic strategies for the treatment of HFpEF.

One possible treatment strategy counteracting the fibrotic process and potentially reversing cardiac fibrosis is to inhibit the activation of human cardiac fibroblasts (HCF), the main effector cells of cardiac fibrosis, thereby lowering ECM deposition. We previously identified two natural compounds, bufalin (toad exudate and component of the traditional Chinese medicine “ChanSu”) and lycorine (Amaryllidaceae), as potent pharmacological inhibitors of HCF proliferation in vitro and in vivo [42]. Here, we report on similars and derivatives of these lead compounds with improved efficacy and toxicological profiles. Additionally, we herein present novel molecular insight into the compounds’ mechanism of action.

## Methods

### Human cardiac fibroblast (HCF) culture

Cryopreserved human cardiac fibroblasts (HCF) of multiple donors were obtained by Promocell, Germany (#C-12375). Initial cryovial was thawed and cells were transferred into pre-warmed fibroblast growth medium (FGM-3): 1 ng/mL Basic Fibroblast Growth Factor (bFGF) and 5 µg/mL Insulin (Promocell, #C-39350), 1% Penicillin–Streptomycin (P/S, Gibco, #15140122), 10% Fetal Bovine Serum (FBS, Gibco, #10270106) in Fibroblast Basal Medium 3 (Promocell, #C-23230). FGM medium was exchanged 24 h after thawing and/or 96 h after passaging, cells were passaged every 7 days by washing twice with DPBS and subsequent incubation with Trypsin/EDTA for 3–5 min. 10% FCS in DMEM was used to block trypsin before centrifugation at 300*g*, 4 °C for 5 min.

### Human-induced pluripotent stem cell-derived cardiomyocyte (hiPS-CM) culture and differentiation

Human-induced pluripotent stem cells (iPSC) [19] were cultured and differentiated into cardiomyocytes following the method of Lian et al*.* [29], adapted as described before [6, 14]. Briefly, iPSCs were maintained on Geltrex (Gibco, #A1413302) in SC medium (StemMACS iPS-Brew XF with supplement, Miltenyi Biotec, #130-104-368) and passaged on confluency with Versene (Gibco, #15040066) + 2 µM Thiazovivin (Selleckchem, #S1459) in SC medium.

At a confluency of 70–80%, directed cardiomyocyte differentiation was initiated by incubation with 250 mg human recombinant albumin (Sigma-Aldrich, #A9731), 100 mg L-AA (L-ascorbic acid 2-phosphate sesquimagnesium salt hydrate, Sigma-Aldrich, #A8960), 5 μM GSK-3 inhibitor XVI (Merck, #361559) in 500 mL RPMI-G medium (RPMI 1640 + GlutaMAX™, Gibco, #72400047) for 48 h. Subsequently, medium was changed and supplemented with 5 mM of the Wnt signaling inhibitor IWP-2 (Selleckchem, #S7085), followed by medium changes with albumin and L-AA in RPMI-G medium every 48 h. From differentiation day 8 on, cells were cultured with 1 × B-27™ (Gibco, #17504001) in RPMI-G, and medium was changed every 2–3 days.

Cardiomyocytes were purified using metabolic selection [47] by culturing the cells for 4–10 days with 4 mM DL-lactate (Merck, #L4263, in 1 M HEPES, Carl Roth, #HN77.3), albumin, L-AA, in no glucose RPMI medium (RPMI 1640, no glucose, Gibco, #11879020).

### In vitro anti-proliferative activity

96-well plates (TPP) were coated with 0.1% gelatine and seeded with 7500 HCFs/well. After 72 h, medium was exchanged with serial dilutions of compounds in FGM-3 + 1.6% DMSO + 1% 5-bromo-2′-deoxyuridine (BrdU, Roche, #11647229001), prepared in additional plates. After 24 h, cells were washed twice with DPBS (Gibco, #14190144) before incubation with anti-BrdU:POD (Roche, #11647229001) for 60 min (RT). Readout was performed by measurement of absorbance at 370 nm + 490 nm in a Synergy HT (BioTek) microplate reader. Reference wavelength-corrected values were analysed using the ECanything function of GraphPad Prism, normalized to respective TOP, to determine EC50 (effective concentration 50%), as well as EC5 and EC95, ± 95% CI (confidence interval). As validation, HCF were seeded as before, but treated with serial dilutions of compounds after 24 h. After further 24 h, medium was exchanged to 10% 2-(4-Iodophenyl)-3-(4-nitrophenyl)-5-(2,4-disulfophenyl)-2H-tetrazolium (WST-1, Roche, #11644807001) in FGM-3. After 60 min of incubation (37 °C, in the dark), absorbance at 450 nm + 630 nm was measured maintaining 37 °C and analysed as described before.

To assess screening quality, quality acceptance criteria Z′ (Z prime), SW (signal window), S:B (signal-to-background), S:N (signal-to-noise) were determined following [20, 55]. Average Z′ ≥ 0.5 and SW ≥ 2 were required to accept the screening overall, whereas single plates were rejected from analysis if Z′ < 0.3 or SW < 1. Compounds were categorized as “active” if *n* ≥ 3 repetitions and EC50 < 10 µM in both assays.

From this point on, we employed the respective 10 × EC50 of each compound for all experiments unless noted otherwise. Importantly, due to the high EC50 of lycorine, we could generate only 2 × EC50 as the highest relative concentration.

### In silico ADME prediction, prediction score and chemical similarity

**Fig. 1:**
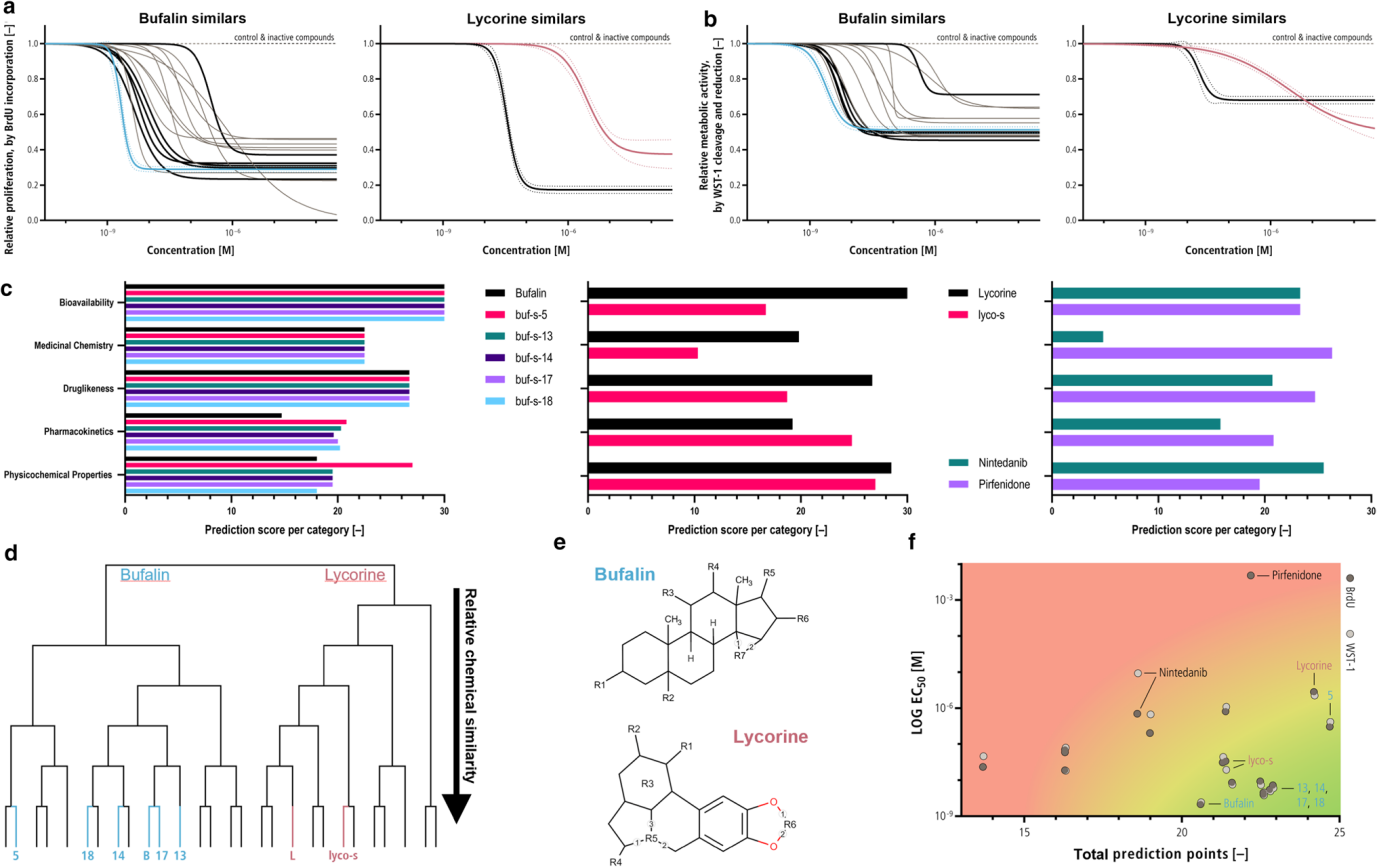
Similars of bufalin and lycorine inhibit human cardiac fibroblast (HCF) proliferation. **a** 15 similars of bufalin and 11 similars of lycorine were investigated for their inhibitory potential on HCF proliferation, determined by BrdU incorporation. 14 similars of bufalin were able to inhibit HCF proliferation in a dose-dependent manner, whereas only one similar of lycorine (lyco-s) showed inhibitory activity. Solid lines represent calculated dose–response for bufalin (blue), lycorine (red), working set (black) and remaining active similars (grey). Dashed line represents control and inactive compounds, and dotted lines show exemplary 95% confidence interval for bufalin (blue), lycorine (red) and lyco-s (black). *n* = 3–7 biological replicates, 6–7 technical replicates each. **b** Similars inhibit metabolic activity of HCF (determined by WST-1 assay) with comparable EC50s as seen for BrdU incorporation. Lines and colours as in **a**. *n* = 4 biological replicates, 3–4 technical replicates each. **c** In silico prediction suggests superior drug properties for bufalin similars of the working set, whereas lyco-s scored lower than lycorine in most categories. The anti-fibrotic drugs nintedanib and pirfenidone (used in idiopathic pulmonary fibrosis, IPF) are in a comparable range for most categories. **d** Analysis of chemical structure showed high similarity between bufalin and the chosen similars, but not lycorine and lyco-s. **e** Sites of modification of bufalin and lycorine similars. **f** Working set of similars was selected by combining BrdU and WST-1-derived EC50 with the in silico-derived prediction score. Bufalin, lycorine, and similars selected in the working set are highlighted. Approved IPF drugs nintedanib and pirfenidone are shown for comparison

After SMILES (string representation of molecular formula using the “Simplified molecular-input line-entry system”) were determined for every compound using *Marvin* (ChemAxon), additional properties were predicted in silico using *SwissADME* [10]. For all parameters, compounds were scored as negative/poor (0) to positive/optimal (30). Weighted scores were grouped into the categories physicochemical properties, pharmacokinetics, drug‐likeness, medicinal chemistry, as well as bioavailability. Weighted category averages (2, 3, 2, 1, 1, respectively) were used to determine an average prediction score from 0 (poor) to 30 (optimal prediction) for every compound. Top 6 candidates were chosen by combining low EC50 with high prediction score (Fig. 1f), as well as the 2 original lead compounds for comparisons. Chemical similarity was determined by calculating Tanimoto coefficients of the fingerprints using *C-SPADE* [38].

### In vitro toxicity

As contrast to activity, toxicity of top six similars and lead compounds was determined by a multiplex measurement of caspase activation and membrane integrity (using LDH release and inclusion of the CellTox Green dye). Briefly, HCFs were seeded as before. After 24 h, cells were treated with serial dilutions of compounds with additional 0.1% CellTox™ Green dye (Promega, #G8741). After 24 h incubation, 5 µL medium was removed from each well, mixed with 95 µL LDH Storage buffer (Promega, #J2380) and frozen for later analysis. 95 µL Caspase-Glo® 3/7 reagent (Promega, #G8090) was added to each well, and after 60 min of incubation luminescence was measured in a Synergy HT. Afterwards, micrographs of brightfield and green fluorescence were taken using a Cytation 1 (BioTek) automated microscope maintaining 37 °C and 5% CO_2_. Wells were manually checked for co-localization of fluorescence signal with cells in brightfield. On the next day, supernatants in LDH Storage buffer were thawed, and 50 µL was mixed with 50 µL of LDH Detection reagent and luminescence was measured after 60 min of incubation. Dose–response curves were determined as described before. To determine toxicity of the top 3 anti-fibrotic similars in hiPS-CM, 20,000 cells/well were seeded, treated and analysed as above, devoid the fluorescent dye steps.

### Ex vivo toxicity in human living myocardial slices

Myocardial slices were generated from human left ventricular heart specimens taken from failing hearts at the time of transplantation. Tissue was obtained from the Clinic for Cardiac, Thoracic, Transplant and Vascular Surgery at the Hannover Medical School (MHH), Hannover, Germany. Patients provided informed consent to the scientific use of the explanted tissue. The study was performed in accordance with the ethical standards laid down in the 1964 Declaration of Helsinki and its later amendments.

The preparation [16, 50] and culture [13] of myocardial slices has been described before. Briefly, human heart specimens were sliced into 300 µm thick slices using a vibrating microtome (Campden Instruments, #7000smz-2) while in ice-cold 30 mM 2,3-butanedione monoxime + 1 mM d-glucose + 10 mM HEPES + 6 mM KCl + 140 mM NaCl + 1 mM MgCl_2_ + 1.8 mM CaCl_2_ (cardioplegic Tyrode’s solution), before being trimmed into approx. 7 × 7 mm pieces of aligned muscle fibres. Subsequently, pieces were attached to plastic rings (3D-printed in-house) using Histoacryl® (B. Braun, #1050052) and transferred into specifically designed culture chambers (mechanically stretched to a sarcomere length of 2.1 µm, continuous electrical stimulation at 0.2 Hz, 20–30 mA, 3 ms) filled with 0.1% DMSO or compound in Medium 199 (Sigma-Aldrich, #M4530).

After 48 h in culture, myocardial slices were frozen in liquid nitrogen and stored at −80 °C for further gene expression analysis.

### In vitro migration assays

To assess migrative capabilities of HCF, cells were stained with 1:1000 1,1′-Dioctadecyl-3,3,3′,3′-Tetramethylindotricarbocyanine Iodide (DIR′) dye in FGM-3 for 20 min at 37 °C. Cells were washed with DPBS and seeded into 0.1% gelatine-coated 96-well plates at 30,000 cells per well. After 24 h incubation, scratches were introduced manually using a small pipette tip, cells were treated with 10 × EC50 of the respective compound, and plates were imaged using an Odyssey Imager. After 17 h and 24 h, plates were imaged again. Rate of migration was determined using the migrative index (MI) [31].

### Real-time qPCR and overexpression/inhibition

Cells were collected in QIAzol Lysis Reagent (Qiagen, #79306) for RNA isolation via precipitation method or with miRNeasy Mini Kit (Qiagen, #217004). Reverse transcription of mRNA was performed with iScript Select cDNA Synthesis Kit (Bio-Rad, #170-8897) using oligo dT primers. cDNA was diluted 1:3 with dH_2_O prior to the real-time qPCR with iQ SYBR Green Supermix (Bio-Rad, #1708882), including ROX Reference Dye. A mix of forward and reverse primer pairs (10 µM, Eurofins) or 10 × Quantitect Primer Assay (Qiagen, #249900) was used for mRNA. To measure miRNA, RNA was reverse-transcribed using the miRNA TaqMan MicroRNA Reverse Transcription Kit (Applied Biosystems, #4366597) with specific miRNA RT primers (ThermoFisher, #4427975). cDNA was diluted 1:3 with dH_2_O before qPCR with Absolute Blue qPCR Mix (Abgene, #AB-4136/B) and the specific Taqman probes (ThermoFisher, #4427975). Runs were performed in ViiA7 (Applied Biosystems) or QuantStudio 7 Flex (Applied Biosystems). Real-time qPCR data were analysed as described elsewhere [30].

### miRNA array and mRNA panel determination

For assessment of miRNA deregulation, TaqMan® Array Human MicroRNA Cards (754 miRNAs, Applied Biosystems™, #4444913) were used according to the manufacturer’s instructions. Briefly, 590 ng of isolated total RNA of HCF (2 individual replicates for each of 3 different passages) was mixed with the Megaplex Primer Pool (A or B) and reverse-transcribed as instructed. cDNA was gently mixed with TaqMan® Universal Master Mix II, no UNG (Applied Biosystems™, #4440043) and dH_2_O and loaded onto the cards. PCR amplification of array cards was performed as instructed. After quality control of all real-time qPCRs, cT values were exported and all runs were merged into a single excel file. Recommended baseline correction using relative thresholds to correct for varying reconstitution rates of different Taqman probes was not performed. Low-expressed miRNAs with cT > 32 in more than 1 replicate per treatment were excluded from analysis, before samples were normalized to column mean. Multiple unpaired *t* tests were performed using GraphPad Prism, deregulations were accepted as discoveries if FDR ≤ 1% (using BKY adjustment [5]). As miRNA identifiers were based on miRbase v14, we converted them to the latest v22 for further analysis using *miRBaseConverter* [54]. 747 sequences could be converted correctly, while the remaining miRNAs were removed from miRbase due to incorrect annotation (e.g. the sequences of hsa-miR-886-3p and hsa-miR-886-5p are part of VTRNA2-1 [44]) or no evidence of human expression.

All discoveries from bufalin and its similars were grouped as “bufalin-like”, and discoveries from lycorine and lyco-s as “lycorine-like”, for pathway enrichment using *miRNA Enrichment Analysis and Annotation* (miEAA v2.0, [21]). Over-Representation Analysis (ORA) was performed using all converted sequences as reference set, and for ORA and miRNA enrichment analysis ((G)SEA) pathways were deemed significant if BH [3] adjusted *p* value < 0.05 and at least 2 hits per subcategory. The 7 most deregulated miRNAs were further validated by real-time qPCR and overexpression/inhibition analysis.

To determine whether our compounds were effective in fibrotic signalling, we used the nCounter® Fibrosis Panel (nanoString, #XT-CSO-HFIB2-12), which allows measurement of 770 mRNAs related to 4 different stages of fibrosis, according to the manufacturer’s instructions. Briefly, 100 ng/5µL isolated total RNA of HCF after 24 h treatment (RIN > 7.5 and DV300 > 88%) was mixed with the hybridization Master Mix and incubated at 65 °C for 20 h before loading on a nCounter® FLEX (Department of Pathology, Hannover Medical School, Germany). All run files (.RCC) were compiled into a single experiment using the *nSolver* analysis software. After quality assessment with the R package *NanoString QC Pro* (v1.22, [34]), we excluded 1 sample (medium control of Lot 5) for inconsistent count data. Reference genes for normalization were determined by *nSolver* using all samples (ACAD9, ARMH3, CNOT10, GUSB, MTMR14, NOL7, NUBP1, PGK1, PPIA, RPLP0, see supplemental Fig. 4e, f). We then used the *Advanced analysis* plugin (v2.0.115) including LOT as confounder variable and BY adjusted *p* value < 0.05 for identification of central deregulated pathways. Additionally, normalized counts were further analysed with GraphPad Prism using multiple unpaired *t* tests with FDR ≤ 5% after BKY adjustment [5] and log2 |FC| ≥ 1. *DiVenn* was used to identify the shared deregulated genes after treatment with similar compounds (bufalin-like or lycorine-like), as well as after treatment with lyco-s in multiple culture conditions (data not shown) [45].

### Global RNA sequencing

The NEBNext Ultra II Directional RNA Kit was used to prepare the samples (the same as for the nCounter® Fibrosis Panel, see above) for subsequent deep sequencing on a NovaSeq PE50 (Illumina) at HZI Braunschweig (Germany). Reads were trimmed and mapped to the human genome hg38.79 using RNA *STAR* (v2.4.2a, [11]) by HZI Braunschweig. RAW read counts were further analysed on *Galaxy* (v3.38.3, usegalaxy.org, [2]) using the *limma-voom* method [26, 43]. Low-expressed transcripts without more than 0.25 CPM (counts per million reads mapped) in at least 3 samples (42,079 of 61,043) were filtered out, and TMM (trimmed mean of M values) was the method used to normalise library sizes. Changes in transcript expression were considered significant if FDR [4] ≤ 0.05 and log2 |FC| ≥ 1.3. GSEA was performed using *enrichR* [7, 24], combined as well as separate for up- and down-regulated genes.

### Statistical analysis

Statistical analyses were performed with GraphPad Prism (versions 7, 8, and 9) or as described in the other methods. Generally, unpaired Student’s *t* tests were used to compare two groups, one-way or two-way ANOVA (as mixed model if values were missing) to compare multiple groups. Findings were deemed significant if *p* value < 0.05 (*), 0.01 (**), 0.001 (***), 0.0001 (****). *p* values were adjusted for multiple comparisons with Dunnett/Tukey, BH (Benjamini–Hochberg), BY (Benjamini–Yekutieli), or BYK (Benjamini–Krieger–Yekutieli) whenever appropriate. Values with *Q* = 0 were removed from Volcano plots for graphing reasons. Several heatmaps were created using the clustergram function of MATLAB (versions 2019a, and 2020a) exported using the “export_fig” plugin (version 3, Y. Altman, https://github.com/altmany/export_fig).

## Results

### Identification of anti-fibrotic efficacy of natural compound similars

We previously identified anti-fibrotic activities of bufalin and lycorine in a screen of 480 natural compounds [42]. To search for compounds with even further improved anti-fibrotic properties, we now analysed a selection of “similars”, small molecules closely related to these lead substances. In a first step, we investigated whether these similars demonstrate potential to reduce cardiac fibroblast proliferation (Fig. 1a). Activity of similars on human cardiac fibroblasts (HCF) was determined by BrdU incorporation (first screen) and WST-1-derived metabolic activity (second screen). Overall, 14 out of 15 similars of bufalin (buf-s-*n*) showed anti-proliferative effects (EC50 < 10 µM) with EC50s in the range of 5–500 nM (Fig. 1a). In comparison, only 1 out of 11 similars of lycorine could reduce HCF proliferation. When compared to lycorine, the EC50 of this similar, from here on termed “lyco-s” (lycorine similar, later identified as homoharringtonine), was determined to be over 100-fold lower than the EC50 of the lead substance lycorine. The EC50s determined by BrdU incorporation could be validated by WST-1-derived metabolic activity for all compounds (Fig. 1b) with 0.04 ± 0.19 log units difference between BrdU and WST-1-derived EC50s. Please note differences between the compounds observed on the *Y*-axis do not necessarily translate to metabolic differences due to assay variability.

In addition to these efficacy measurements, we predicted physicochemical properties, pharmacokinetics, drug‐ and lead‐likeness, as well as bioavailability in silico using the *SwissADME* webservice [10]. All compounds were ranked in each category, leading to an “ADMET” prediction score ranging from 0 (unfavourable) to 30 (optimal in all categories) (Fig. 1c). This score was used as an additional ranking dimension to determine our working set of the 2 initial lead compounds and 6 follow-up similars for further follow-up studies (Fig. 1f). When compared to nintedanib and pirfenidone, the two leading anti-fibrotic drugs approved for treatment of idiopathic pulmonary fibrosis, our newly identified natural compound similars show higher activity and similar or better prediction scores.

To assess the striking differences in bufalin and lycorine similars, we first compared the chemical structures (Fig. 1e, working set of compounds highlighted). We could observe a high similarity between bufalin and the chosen similars buf-s-13, buf-s-14, buf-s-17 and buf-s-18, mostly differing in a single bond only (Fig. 1d). Only bufalin similar buf-s-5, chosen for its high prediction score, exhibits notable differences in structure. Surprisingly, the only active lycorine similar lyco-s was not as closely related to the lead substance as expected, but had an additional side chain and a differing scaffold approximately doubling its molecular weight.

### Toxicological assessment and functional validation of natural compound similars

**Fig. 2:**
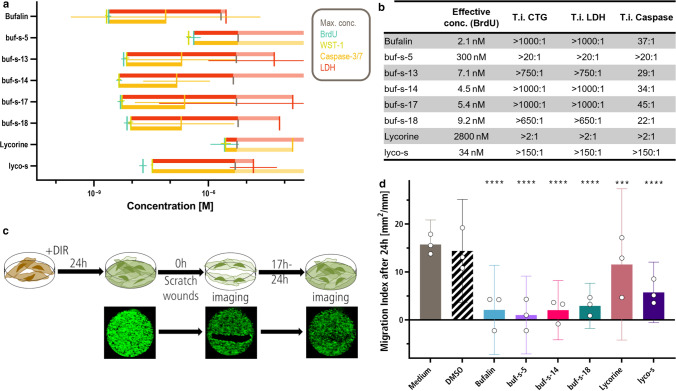
Favourite similars buf-s-14, buf-s-18 and lyco-s have low toxicity and inhibit HCF migration. **a** In addition to efficacy (BrdU, teal; WST-1, green), we determined cytotoxicity via dye inclusion (CellTox Green, not shown), LDH release (red) as well as Caspase-3/7 activation (yellow). Vertical lines represent EC50, horizontal lines 95% CI. Horizontal bars indicate therapeutic index (T.i., IC50 ∕ EC50) until LDH (red) or caspase activation (yellow). Pale part of bars indicates predicted range above the highest tested concentration. *n* = 3 biological replicates (donors), 2 technical replicates each. **b** Effective concentration (EC50 by BrdU assay) and T.i. (IC50 / EC50) for working set. CTG: CellTox Green dye. **c**, **d** HCF were stained with 1:1000 DIR before plating. After 24 h incubation, cells were scratched and subsequently treated with the compounds (**c**). All compounds were able to inhibit HCF migration (**d**). *n* = 3 biological replicates, six technical replicates each. Bars represent mean ± 95% CI. Analysed with Two-way ANOVA (*p* < 0.05), adjusted following Dunnett. DIR: 1,1′-Dioctadecyl-3,3,3′,3′-Tetramethylindotricarbocyanine Iodide

After we demonstrated anti-proliferative activity of selected similars, we assessed potential cytotoxic effects by measuring caspase activation and membrane integrity (using LDH release and inclusion of the CellTox Green dye) to rule out that anti-fibrotic effects were mediated mainly by cell death. Comparison of efficacy (BrdU) and cytotoxicity measurements yielded therapeutic indices (T.i., ratio of toxic to effective concentration) for each compound of the working set (Fig. 2a, b and Supplemental Fig. 2a). We did not detect any increase of LDH release or cellular inclusion of CellTox Green dye, indicating persistent membrane integrity after compound treatment. Treatment with bufalin, buf-s-13, buf-s-14, buf-s-17, and buf-s-18 led to dose-dependent induction of Caspase-3/7 within the tested concentration range. Most probably, true EC50s for cytotoxicity (Fig. 2a, red crosses) as well as Caspase induction (Fig. 2a, yellow crosses) are at higher concentrations than tested, placing T.i. for most compounds well over 1000:1 and likely higher than noted in Fig. 2b.

Furthermore, we treated human iPS-derived cardiomyocytes (hiPS-CM) with selected compounds (Supplemental Fig. 2b–e). hiPS-CM showed neither increased Caspase activation nor LDH release after treatment at effective concentrations, but we observed increased cytotoxicity at maximal available concentrations (Supplemental Fig. 2d, e). This led to smaller, but still useable T.i. compared to the treatment of HCF (Supplemental Fig. 2b, c). Furthermore, we employed human living myocardial slices, an ex vivo model of human left ventricular heart tissue under constant electrical stimulation ([23, 51], details see methods section). qPCR analysis revealed significant down-regulation of FAP (supplemental Fig. 2g, Fibroblast activation protein alpha) and MMP2 (supplemental Fig. 2j) after treatment with lyco-s, but not lycorine.

As our working set of compounds overall showed favourable safety at effective concentrations in HCFs and hiPS-CM, we investigated further functional anti-fibrotic properties. HCF treated with selected bufalin similars and the lycorine similar showed markedly impaired fibroblast migration (Fig. 2c, d). Interestingly, using microscopic evaluation after treatment with bufalin or its related similars (buf-s-5, buf-s-14, buf-s-18), we observed nearly complete lack of migration within the first half hour. In contrast, treatment with lyco-s repressed migration only after several hours, explaining the slightly elevated migration index. In summary, the two lead compounds as well as the chosen similars are able to impair fibroblast proliferation and migration.

### Natural compound similars regulate a set of fibrosis-associated miRNAs

**Fig. 3:**
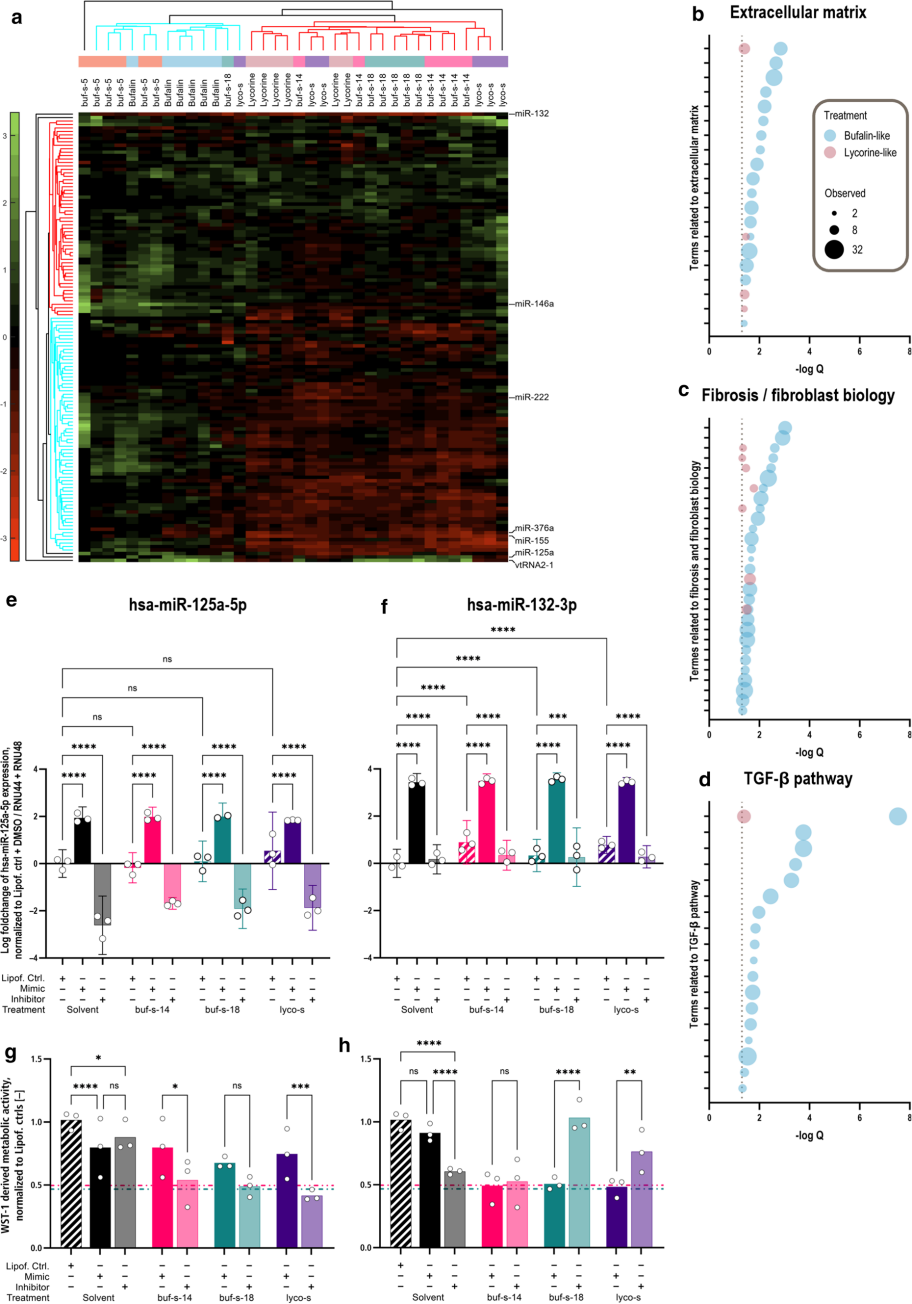
Natural compound similars regulate a set of fibrosis-associated miRNAs. **a** Treatment with compounds led to upregulation of several miRNAs across all treatments, whereas downregulation of a set of miRNAs was observed in buf-s-14, buf-s-18, lycorine as well as lyco-s. miRNAs analysed in more detail indicated on the right. *n* = 3 biological replicates (donors), 2 technical replicates each. Discoveries determined with multiple unpaired *t* tests, adjusted following BKY (*Q* = 0.01). Pathways of significantly deregulated miRNAs, summarized by “bufalin-like” (blue) or “lycorine-like” (red) treatment, were determined by over-representation analysis (ORA) using miEAA2, adjusted following BH (*Q* = 0.05). A high number of terms were found related to extracellular matrix (**b**), fibrosis and fibroblast biology (**c**) as well as TGF-β pathways (**d**). As the analysis included more similars of bufalin than lycorine, more significant terms were observed for “bufalin-like” treatment overall. Dotted line indicates significance threshold (*p*_adj._ < 0.05). Real-time qPCR analysis showed hsa-miR-125a-5p expression was independent of compound treatment (**e**), whereas hsa-miR-132-3p was upregulated in compound-treated HCF (**f**). *n* = 1 donor, 3 biological replicates. Analysed with Two-way ANOVA, adjusted following Tukey. Utilizing the WST-1 assay, overexpression of hsa-miR-125a-5p partially protected against the anti-proliferative effect of buf-s-14, buf-s-18 and lyco-s (**g**). In contrast, inhibition of hsa-miR-132-3p protected against buf-s-18 and lyco-s, but not buf-s-14 (**h**). Bars show Lipofectamine control (hatched), overexpression with respective miRNA mimic (full colour) and inhibition with respective miRNA inhibitor (pale colour). Horizontal lines represent remaining activity after treatment with buf-s-14 (pink, dash-dot-dot) and buf-s-18 (teal, dash-dot) without miRNA mimic or inhibitor. *n* = 3 biological replicates (donors), 3–10 technical replicates each. Analysed with Two-way ANOVA (*p* < 0.05), adjusted following Tukey

As the importance of miRNAs in organ fibrosis [32, 46] and cardiovascular disease [1, 22] has been reported, we speculated on the importance of miRNAs regulated through our newly identified anti-fibrotic natural compound similars. An overview of all 165 in HCFs detectable miRNAs is shown in Fig. 3a. We found a common set of miRNAs down-regulated after treatment with buf-s-14, buf-s-18, lycorine and lyco-s, but not bufalin and buf-s-5, whereas a smaller number of miRNAs were upregulated across all treatments. Overall, we identified 43 miRNAs with *Q* value > 2.

We further performed over-representation analysis (ORA) of significantly deregulated miRNAs using the miEAA2 tool [21] (see Supplemental File 1). Briefly, miEAA2 tests whether a given list of miRNAs (without prior conversion to mRNA targets) is significantly over-represented in certain biological pathways, compared to a random selection of miRNAs. Enriched terms (with *q*_adj._ < 0.05) were indeed related to ECM (Fig. 3b), e.g. extracellular matrix structural constituent (GO:0005201, adj. *p* = 5.5 × 10^−3^), extracellular matrix organization (GO:0030198, adj. *p* = 1.3 × 10^−2^), or cell matrix adhesion (GO:0007160, adj. *p* = 2.2 × 10^−2^), as well as fibrosis (Fig. 3c), e.g. regulation of cell migration (GO:0030334, adj. *p* = 1.2 × 10^−3^), regulation of cell proliferation (GO:0042127, adj. *p* = 1.2 × 10^−2^), or fibrosis (MNDR,^1^ adj. *p* = 2.8 × 10^−3^). Additionally, miRNAs controlling the TGF-β pathway were significantly affected (Fig. 3d), e.g. positive regulation of transforming growth factor beta receptor signaling pathway (GO:0030511, adj. *p* = 3.6 × 10^−4^). In summary, our compounds deregulate miRNAs mainly involved in fibrotic processes.

Of the 43 significantly deregulated miRNAs, we chose the top 7, based on absolute fold-change, for further validation and assessment. As such, we were able to validate the significant regulation of miRNA expression by real-time qPCR analysis (exemplarily shown in Fig. 3e, f). To determine whether these miRNAs are part of and essential to the mechanism of action of the compounds, we transfected HCF with miR mimics or inhibitors of the miRNA candidates prior to compound treatment. If a miRNA is essential for the compounds’ mechanism, we would expect the absence of anti-proliferative effects after inhibition of this miRNA, whereas anti-proliferative effects would remain if the miRNA is rather a downstream element of the compound response. Indeed, we were able to detect such an absence of the anti-proliferative effect for hsa-miR-132-3p after treatment with buf-s-18 and lyco-s, but not buf-s-14 (Fig. 3h). Meanwhile, we observed an inverse correlation for hsa-miR-125a-5p, as overexpression of the miRNA was able to prevent the anti-proliferative effect of treatment with buf-s-14 and lyco-s (Fig. 3g). These findings indicate the contribution of these two miRNAs in the signalling required for the anti-proliferative action of the respective anti-fibrotic drug candidates. The remaining miRNAs showed changed expression after compound treatment via real-time qPCR (data not shown), but no significant influence on HCF proliferation could be detected regardless of the treatment (Supplemental Fig. 3c–g).

### Identification of regulated anti-fibrotic pathways

To test a potential influence of newly identified natural compound similars on pro-fibrotic pathways, we utilized a targeted fibrotic signalling mRNA profiling tool. Quality control of all runs led to exclusion of 1 sample (HCF in medium only, Donor 5, Supplemental Fig. 4a–c). Results from the remaining 29 samples were analysed by correlation analysis, indicating relatively high consistency between measurements (Pearson *r* > 0.84 for all comparisons). Moreover, 10 of the genes were chosen as reference genes (Supplemental Fig. 4e, f). Highest-expressed genes after normalization were CXCL8, FN1, and VIM, as well as SERPINE1 in lyco-s-treated samples (Supplemental Fig. 4g). As we observed significant differences between the HCF donors (Supplemental Fig. 4h), we corrected for donor variability as a covariate in the following in-depth analyses.

**Fig. 4:**
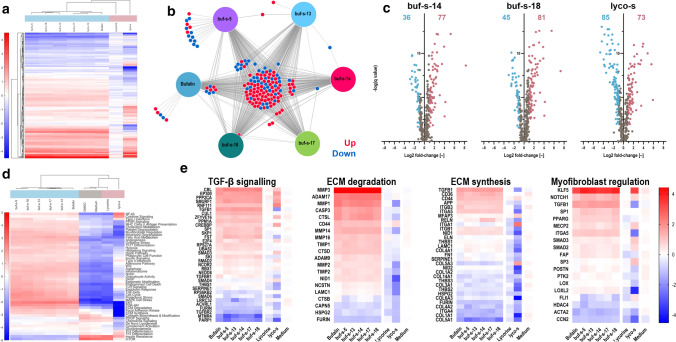
Similars regulate fibrotic mRNA signalling. **a** RNA isolated from HCF treated with bufalin, buf-s-5, buf-s-13, buf-s-14, buf-s-17, buf-s-18, lycorine or lyco-s was analysed using the nanoString Fibrosis panel. Out of 770 mRNAs measured, expression of 369 genes was significantly deregulated and distinct expression profiles for the compound groups bufalin-like (blue) or lycorine-like (red) were obtained. *n* = 3 biological replicates (donors). Analysed with multiple *t* tests (*p* < 0.05), adjusted following BY. **b** 93 genes were up-regulated, respectively, downregulated in all bufalin-like compound-treated HCF, and further 13 genes in at least 5 of the 6 different treatments. **c** buf-s-14 and buf-s-18 tended to increase overall expression, whereas the number of significantly up- and down-regulated genes was similar after treatment with lyco-s. **d** Pathway signatures were scored by average expression of genes in respective pathway, normalized to respective signature mean. Bufalin and its similars have nearly interchangeable pathway profiles, whereas the profile of lycorine is similar to controls. Treatment with lyco-s is the only condition with reduction in collagen, ECM and EMT pathways. **e** Average expression per gene for selected pathways. *n* = 3 biological replicates (donors)

Out of the remaining 760 mRNAs of this targeted panel, 369 showed significant deregulation (BY adjusted *p* value < 0.05) in at least one condition. No significant differences were found between DMSO and medium only control samples. An overview of all significantly deregulated genes is shown in Fig. 4a. Of note, treatment with lycorine resulted in less deregulation (fold-change) compared to the other treatments. Samples treated with lyco-s trended towards more down- than upregulation, and the pattern of up- and down-regulated genes was different from the pattern observed for the remaining treatments. In contrast, bufalin and all bufalin similars shared a highly similar profile. Indeed, out of 156 differentially expressed genes (DEGs) with log2 |FC| ≥ 1 of bufalin and its similars, a set of 93 genes was always up- or down-regulated across all conditions, and further 13 genes in at least 5 of the 6 different treatments (Fig. 4b). Consequently, Volcano plots were highly similar for bufalin similars, but not lyco-s (Fig. 4c). Pathway analysis revealed significant downregulation of several gene clusters after lyco-s treatment (Fig. 4d). Specifically, lyco-s repressed genes related to endothelial mesenchymal transition (EMT), ECM homeostasis as well as collagen biosynthesis (Fig. 4e).

### Next-generation RNA-Sequencing after anti-fibrotic treatments

**Fig. 5:**
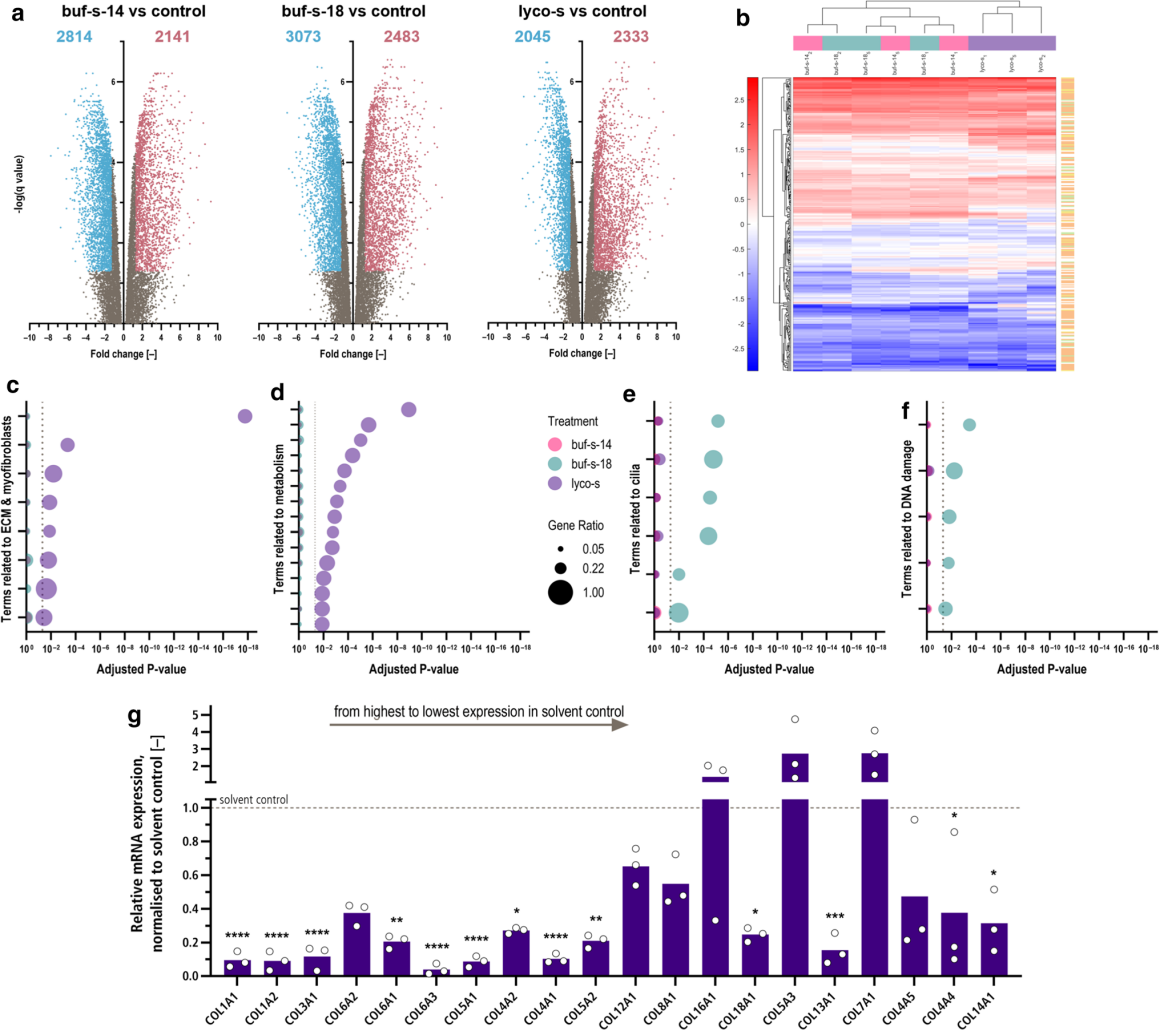
mRNA expression analysis of HCF reveals repression of ECM and collagen function after treatment with lyco-s. **a** Out of 61,043 transcripts, 18,964 remained after exclusion of low-expressed transcripts. Volcano plots show over 4000 differentially expressed genes (DEGs) for all three treatments. Treatment with lyco-s led to a similar amount of significantly up- (red), but not down-regulated genes (blue) compared to bufalin similars. n = 3 biological replicates (donors). Discoveries (*Q* = 0.05) adjusted following BY. **b** Treatment with lyco-s leads to increased deregulation of central pathways related to ECM function, including ECM organisation (orange), collagen binding (green), or both (yellow). GO:BP analysis of DEGs highlights downregulation of ECM and myofibroblast functions (**c**) as well as metabolism (**d**) after treatment with lyco-s. In contrast, treatment with buf-s-18 led to downregulation of genes involved in cilia (**e**) and DNA damage responses (**f**). For samples treated with buf-s-14, no terms were significantly enriched after multiplicity adjustment. Dotted line indicates significance threshold (*p*_adj._ < 0.05). **g** Treatment with lyco-s (purple) repressed most high to medium-expressed collagen isoforms in HCF. The top 5 collagens are among the top 20 overall genes (compare Supplementary Fig. 4d). Mean (bars) and single biological replicates (circles), asterisk indicates significance in Two-way ANOVA (*p* < 0.05), adjusted following Dunnett (including buf-s-14 + buf-s-18, see Supplementary Fig. 4d)

Global transcriptome analysis by RNA sequencing was performed for buf-s-14, buf-s-18 and lyco-s. In total, sequencing detected 61,043 transcripts, of which 42,079 low-expressed transcripts were excluded. Cluster analysis on the remaining 18,964 transcripts could not distinguish buf-s-14 from buf-s-18 (Supplemental Fig. 5a). When compared to DMSO control, we detected over 4000 DEGs for all three treatments (Fig. 5a). We further determined the top 20 expressed transcripts of the DMSO control in more detail to get information about the most important genes of “basal HCF” (Supplemental Fig. 5b). Of note, the 6 highest-expressed transcripts already constitute over 10% of all reads (after filtering, see methods section): FN1 (3.3%), COL1A1 (2.0%), EEF1A1 (1.3%), COL1A2 (1.3%), and two neighbouring lincRNAs. When we assessed the underlying pathways, we found most of those transcripts to be part of pathways expected for fibroblasts (supplemental Fig. 5c): cell–cell adhesion (GO:0034109), ECM organisation (GO:0030198), TGF-beta receptor signaling (GO:0007179) and cell migration (GO:0030334), as well as general protein-related processes (GO:0006414, GO:0044267, GO:0006464).

We further analysed all DEGs of the different treatments using *enrichR* (Fig. 5c–f). For the two bufalin-related similars, down-regulated genes were enriched for terms related to DNA binding, cytoskeleton and multiple pathways of biosynthesis. Analysis of up-regulated genes after treatment with lyco-s enriched terms related to inflammation, whereas down-regulated terms concentrated on ECM, collagen and several biosynthesis pathways.

Of note, ECM organization or ECM-receptor interactions were the top 1 term in GO:BP, KEGG, Reactome and WikiPathways databases. This strong consistency between several databases led us to evaluate the changes in gene expression after treatment with lyco-s in more detail. First, we assessed the gene expression profile of GO biological pathways relevant to ECM and collagen function (Fig. 5b). We detected broad deregulation for many genes related to ECM organisation (GO:0030198, orange) and collagen binding (GO:0005518, green) or both (yellow) after treatment with lyco-s, and to a lesser degree for buf-s-14- and buf-s-18-treated samples. Subsequently, we evaluated all expressed collagen isoforms as central components of fibrosis. From COL1A1 (13.9–14.6 log2 counts) to COL9A1 (− 6.3 to − 4.7 log2 counts), expression level of collagen isoforms spun nearly 6 orders of magnitude. Focusing on the top 20 expressed isoforms, we found significant differences after treatment (ANOVA *p* value < 0.001). As such, treatment with lyco-s led to significantly reduced expression levels of most high- to medium-expressed collagen isoforms, and highest-expressed isoforms were reduced approx. tenfold (Fig. 5g, top 20 collagen isoforms, ordered from highest expressed left to the lowest expressed right). In contrast, collagen levels remained mostly unchanged after treatment with buf-s-14 or buf-s-18 (Supplemental Fig. 5d, same order), as only COL5A1 was downregulated approx. fivefold.

## Conclusion

Here we describe the characterization of molecular similars of the previously identified natural compounds bufalin and lycorine for the treatment of cardiac fibrosis. We selected anti-proliferative candidates based on in vitro screening and in silico predictions, and further validated their effect. In a multi-OMICs approach, we confirmed anti-fibrotic activity and identified several underlying molecular pathways.

The most promising candidate from our screen was lyco-s, also known as homoharringtonine, and we determined EC50 for inhibition of proliferation on HCF at rather low concentrations (34 nM), being in line with the median activity of approved drugs of approx. 20 nM [36] and sufficient to advance further translational developments. Homoharringtonine has been mainly tested as an anti-cancer therapeutic, and has been evaluated in a number of phase II clinical trials (e.g. NCT00375219 [9]). After orphan drug designation in 2006, homoharringtonine has been approved for treatment of chronic myeloid leukemia (CML) by the FDA in 2012.

Historic studies proposed interaction of bufalin with the Na(+),K(+)-ATPase [39] as well as of lycorine and lyco-s with the 80S ribosome [15, 48], recently supported by crystal structures [17, 25]. Notwithstanding, literature reports on the action of bufalin, lycorine and lyco-s are highly inconsistent, often contradictory, and regularly with a relative narrow focus on potential interaction partners or targets. To conclusively resolve anti-fibrotic and selective target profiles of bufalin, lycorine and their similars, we decided on global and unbiased evaluation of the non-coding and coding transcriptome.

To determine changes in miRNA expression, we used a panel of 754 known human miRNAs. We could detect only a minority of 165 miRNAs expressed in HCF, of which 43 were significantly deregulated after treatment. Surprisingly, overexpression of hsa-mir-125a-5p counteracted the anti-proliferative activity of our compounds. Hsa-mir-125a-5p has been described to be a negative regulator of p53, thereby promoting cell proliferation and differentiation [27, 33]. Similarly, increased expression of hsa-mir-125a-5p has been reported in a sheep model of HF [52] and correlated with fibrosis score and severity of hepatic fibrosis and cirrhosis in human [8].

RNA profiling and validation experiments identified an exceptional downregulation by approx. 90% of high- to medium-expressed collagen isoforms after treatment with lyco-s, but not buf-s-14 and buf-s-18. Fibronectin 1 (FN1), Pleiotrophin (PTN), and Biglycan (BGN) are essential markers of myofibroblasts, which act as paracrine signals to other cell populations of the heart [12]. Again, we found all of those factors were significantly downregulated after treatment with lyco-s. Anti-fibrotic effectivity of lyco-s could be validated even ex vivo in human living myocardial slices derived from heart failure patients.

In conclusion, we found strong evidence that the anti-proliferative activity and anti-migratory activity of several of our similars, especially lyco-s, are sufficient to block fibroblast activation and thus validate them as interesting anti-fibrotic drug candidates for further preclinical and clinical development.

## Supplementary Information

Below is the link to the electronic supplementary material.

Supplementary file1 (DOCX 3564 KB)

Supplementary file2 (XLSX 601 KB)

## Footnotes

1 MNDR: mammalian ncRNA-Disease Repository [35].
