# Supplementary material for: Development and characterization of anti-fibrotic natural compound similars with improved effectivity
Source: Basic Res Cardiol. 2022 Mar 2;117(1):9. doi: 10.1007/s00395-022-00919-6 (PMC8891108; doi:10.1007/s00395-022-00919-6)
Supplement: Supplementary file 1 — Supplementary file1 (DOCX 3564 KB) [file 395_2022_919_MOESM1_ESM.docx]

# Supplementary information

## Supplemental figure 1:


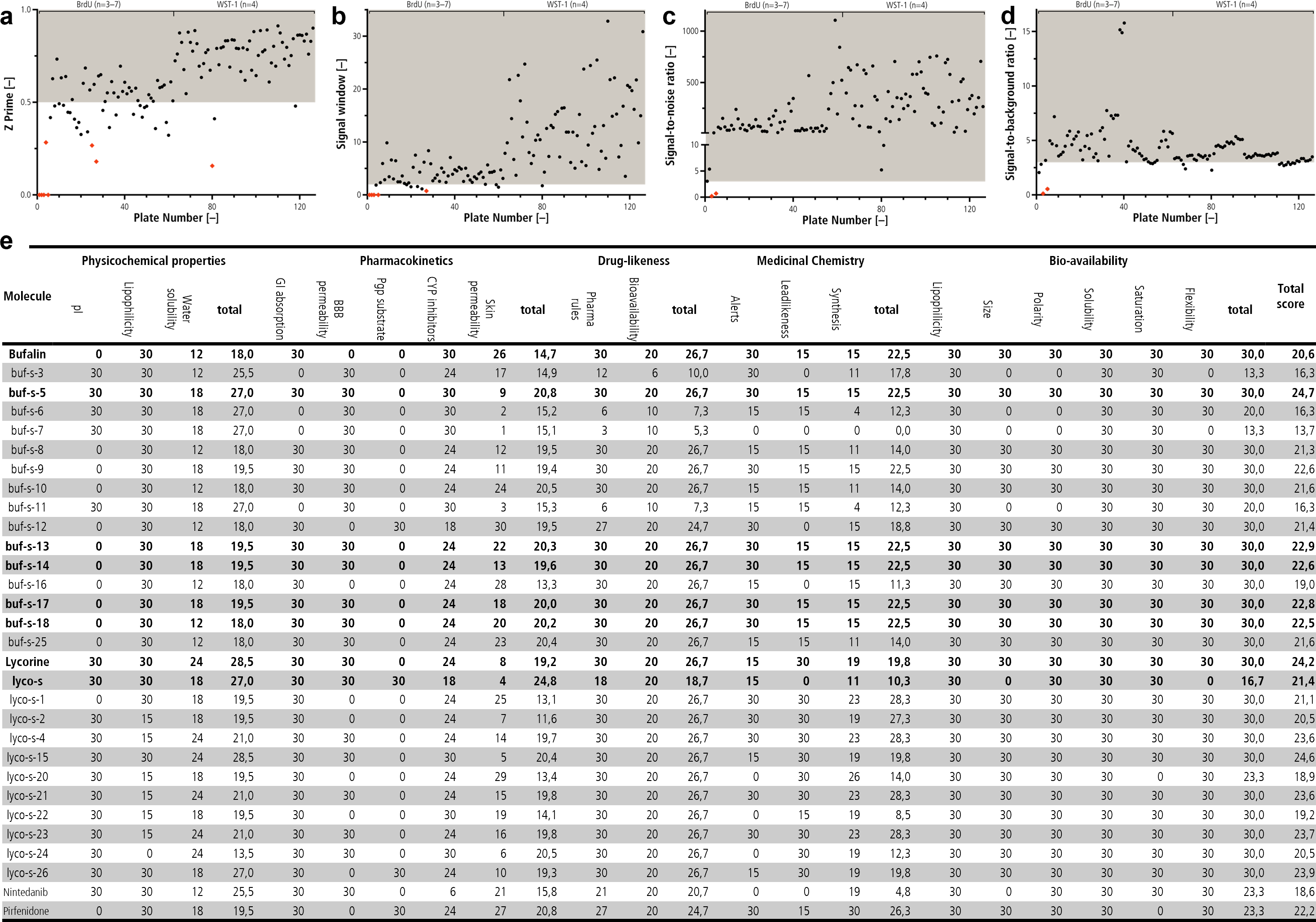


**Supplemental fig. 1**: Screening reliability.

**a**–**d** Quality of first (BrdU) and second (WST-1) screening indicates sufficient reliability between different plates and repetitions. The optimal zone of each parameter, Z′: Z prime (**a**), SW: signal window (**b**), S:N: signal-to-noise (**c**), S:B: signal-to-background (**d**), is highlighted by shading, rejected values are represented by red diamonds. **e** Further parameters were predicted using the SwissADME webservice. Multiple predictions of the same parameter were averaged, before each parameter was scored on a scale from 0 (poor) to 30 (optimal). Scores were weighted and grouped (bold categories), before categories were averaged in a weighted fashion to determine the total prediction score. Similars from the working set are formatted in bold. Please note two-part axis in C.

## Supplemental figure 2:


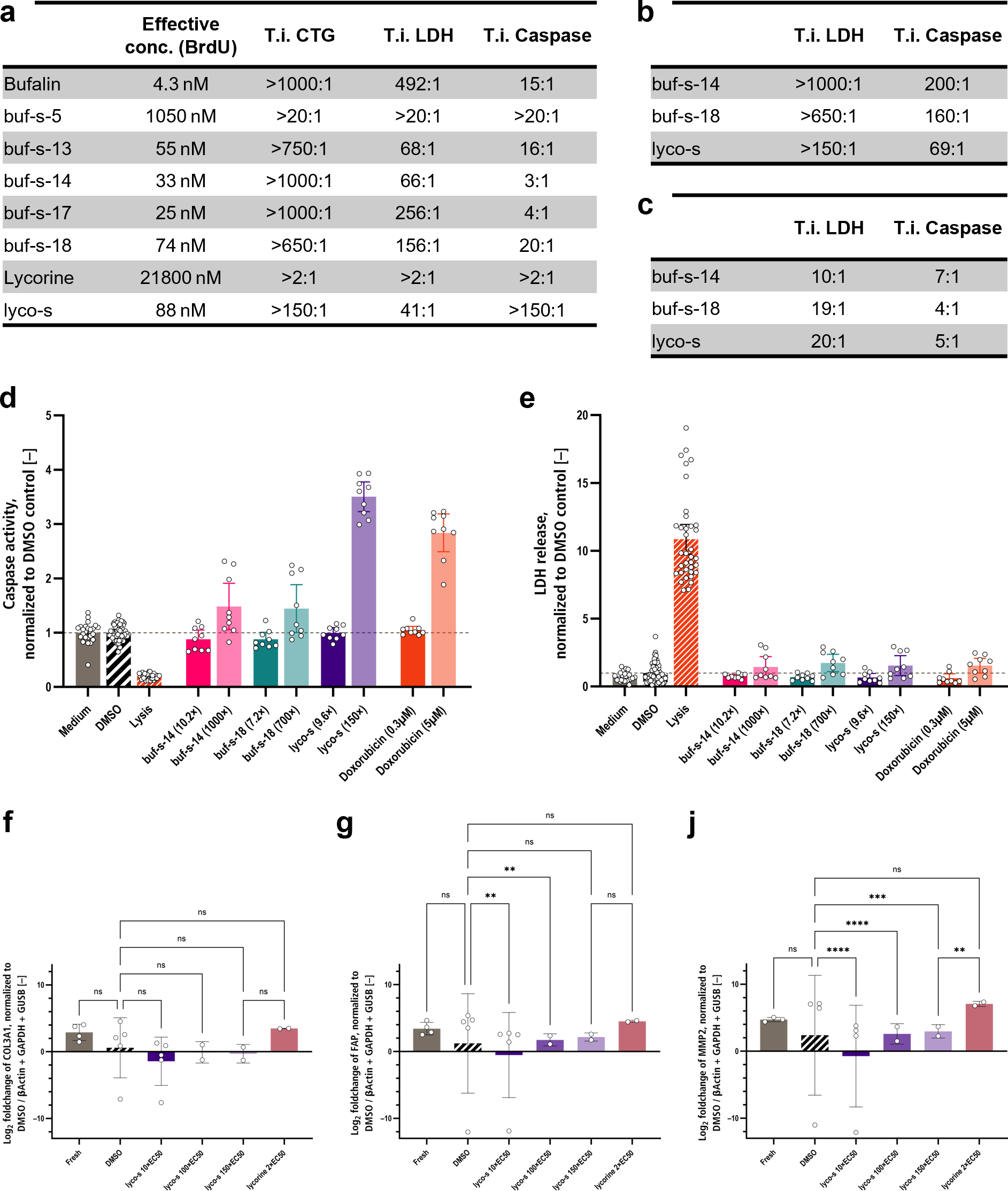


**Supplemental fig. 2**: Favourite similars buf-s-14, buf-s-18 and lyco-s have low toxicity in hiPS-CM and human myocardial slices.

**a** Effective concentration (EC50 by BrdU assay) and strict T.i. (IC5 / EC95) for working set on HCF. **b, c** We determined cytotoxicity in human iPS-derived cardiomyocytes (hiPS-CM) via LDH release and Caspase-3/7 activation, indicating sufficient T.i. (**b**, IC50 ∕ EC50) and strict T.i. (**c**, IC5 / EC95). n=3 biological replicates (differentiations), 3 technical replicates each. **d, e** hiPS-CM are unaffected by effective concentration (approx. 10×EC50 by BrdU assay on HCF) of favourite similars, but show Caspase-3/7 activation (**d**) and increased LDH release (**e**) after treatment with the maximal available concentration for the favourite similars as well as the cardiotoxic chemotherapeutic agent doxorubicin. **f**–**j** Ex vivo human myocardial slices (constant contraction of heart tissue due to electrical stimulation) were treated with lyco-s and lycorine for 48h. qPCR analysis indicated downregulation of FAP (**g**, Fibroblast activation protein alpha) and MMP2 (**j**) after treatment with lyco-s, but not lycorine. 150×EC50 of lyco-s and 2×EC50 of lycorine are comparable molar concentrations. n=2–5 biological replicates (donors), 1–4 technical replicates each. Analysed with Two-way ANOVA (p<0.05), adjusted following Tukey.

## Supplemental figure 3:


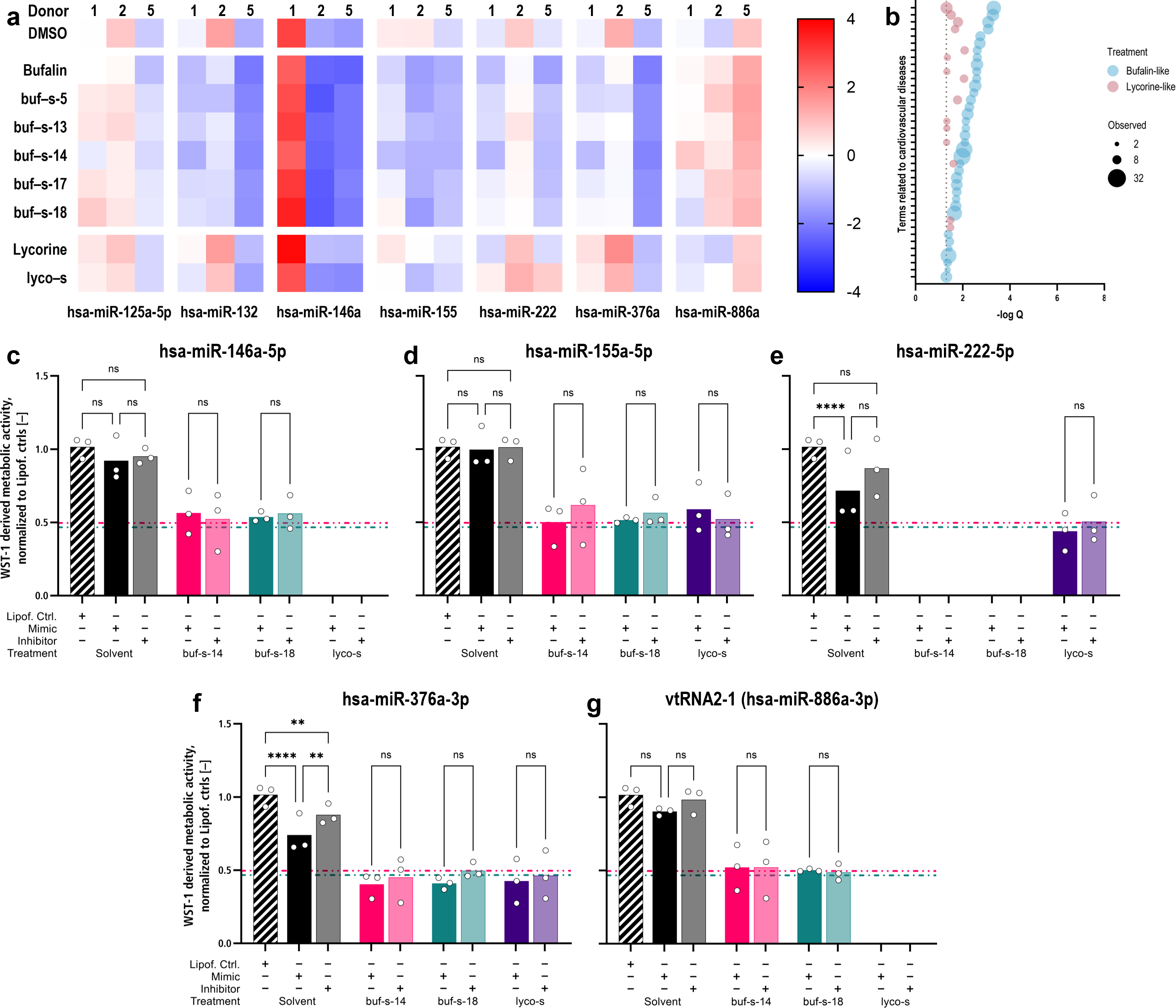


**Supplemental fig. 3**: Donor variability and miRNA overexpression / inhibition.

**a** Out of all significantly deregulated miRNAs identified in the array, we validated the TOP7 in fresh HCF samples using real-time qPCR. Noticeably, miRNA expression levels are highly dependent on HCF donor, masking differential regulation. Still, treatment with bufalin- and lycorine-like compounds led to distinct expression patterns. n=3 biological replicates (donors). **b** Over-representation analysis of significantly deregulated miRNAs using miEAA2 identified several terms related to cardiovascular diseases. Terms related to cardiac contractility were only found after treatment with bufalin-like compounds. Dotted line indicates significance threshold (p_adj._<0.05). **c**–**g** Neither overexpression nor inhibition of hsa-miR-146a-5p (**c**), hsa-miR-155-5p (**d**), hsa-miR-222-5p (**e**), hsa-miR-376a-3p (**f**), or hsa-miR-886a-3p (**g**, also known as VTRNA2-1) protected against anti-proliferative effect of buf-s-14, buf-s-18 and lyco-s. Bars show Lipofectamine control (hatched), overexpression with respective miRNA mimic (full colour) and inhibition with respective miRNA inhibitor (pale colour). Horizontal lines represent remaining activity after treatment with buf-s-14 (pink, dash-dot-dot) and buf-s-18 (teal, dash-dot) without miRNA mimic or inhibitor. n=3 biological replicates (donors), 3–10 technical replicates each. Analysed with Two-way ANOVA (p<0.05), adjusted following Tukey.

## Supplemental figure 4:


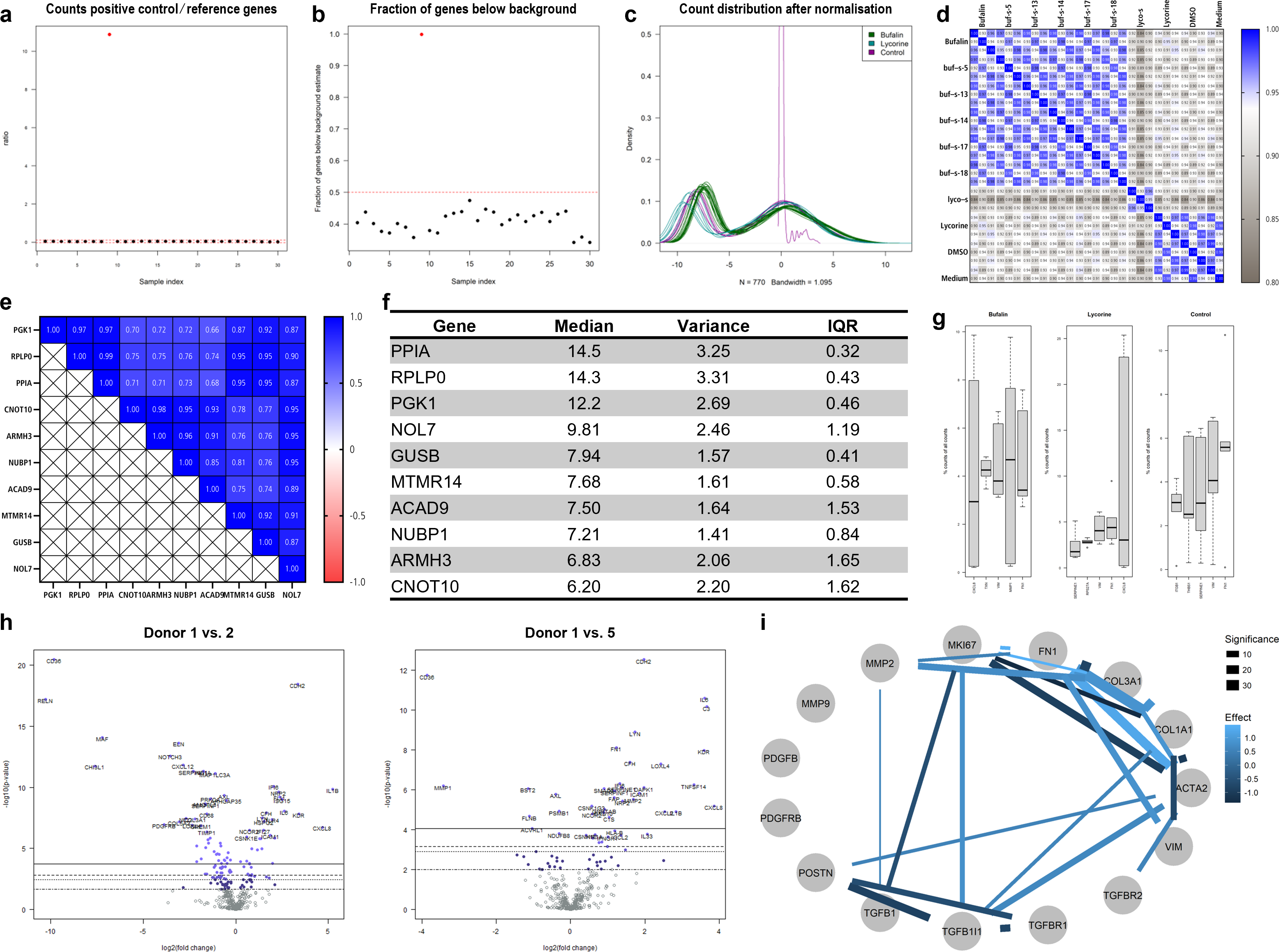


**Supplemental fig. 4**: QC of mRNA array and basal HCF phenotype.

**a**–**c** One sample was excluded due to high signal in assay controls (**a**), but not genes (**b**), resulting in severely skewed count distribution (**c**). **d** Generally, all samples showed high correlation (Pearson r), but still cluster by bufalin and all its similars, lycorine and the controls, as well as lyco-s. Samples from donor 2 show lower correlation across all treatments. Samples ordered by treatment, then HCF donor (1, 2, 5). n=3 biological replicates (donors). **e**, **f** 10 genes, selected as reference genes across low- to high-expressed genes for low variance and interquartile range (IQR), show relatively robust correlation. **g** The TOP5 highest expressed genes in HCF were similar for bufalin and similars, lycorine and lyco-s, as well as control samples, although mean expression was lower in the lyco-s group. **h** Significant differences in gene expression were observed between the HCF donors. Adjusted p value indicated by horizontal lines: < 0.01 (solid), < 0.05 (dashed), < 0.10 (dotted), < 0.50 (dash + dots). **i** We selected a subset of 15 genes commonly used as markers of cardiac fibroblasts. The proliferation marker MKi67 correlates with expression of COL3A1, FN1 and TGFB1I1, suggesting a proliferating HCF phenotype. In contrast, the set of ACTA2, COL1A1, MMP2, POSTN and TGFB1, all common myofibroblast marker genes, inversely correlate with MKi67. This suggests two different subsets of fibroblasts, a proliferating and activated type vs the differentiated myofibroblast type.

## Supplemental figure 5:


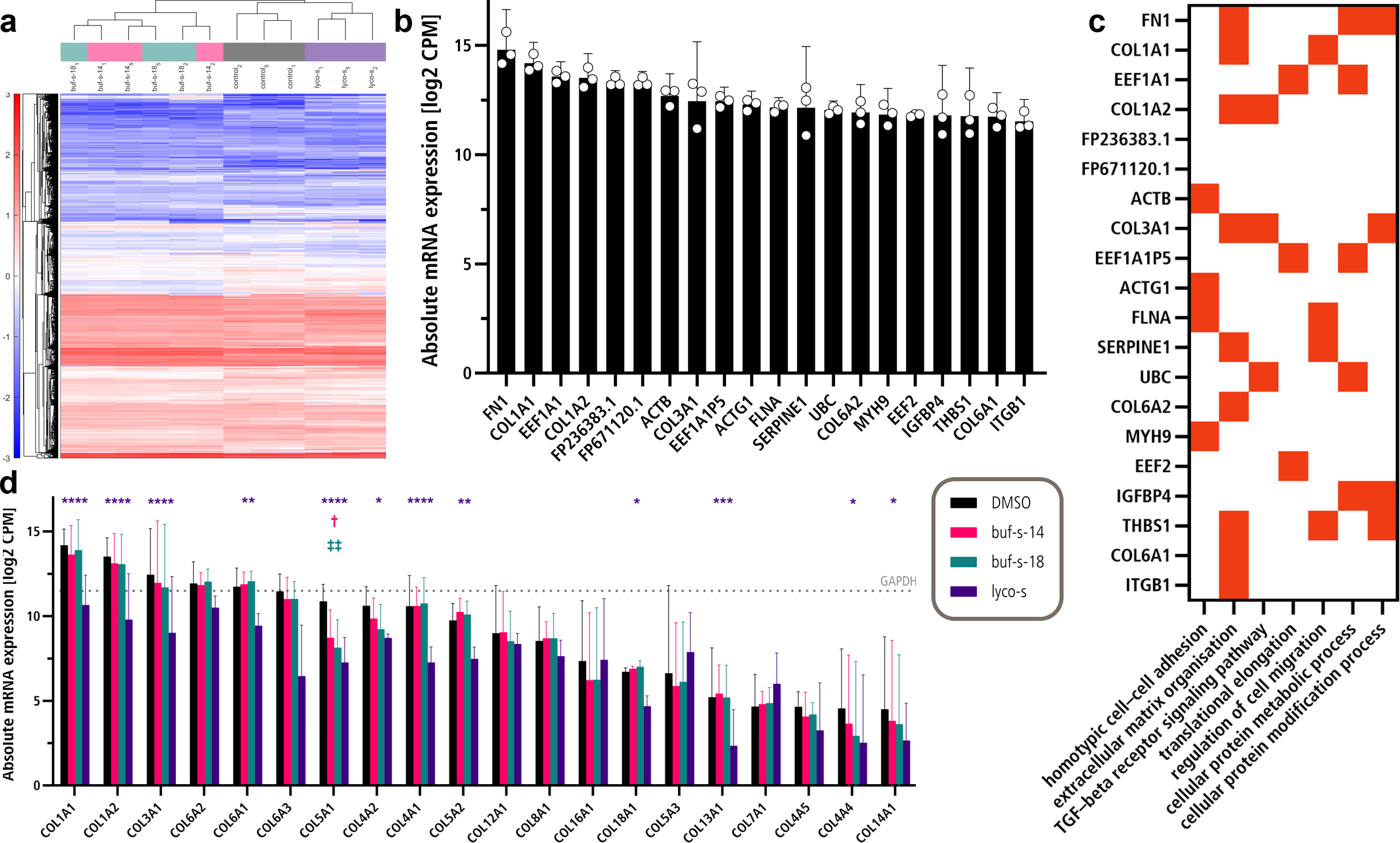


**Supplemental fig. 5**: mRNA-Sequencing of HCF treated with buf-s-14, buf-s-18 and lyco-s.

**a** Over all DEGs, samples cluster by treatment. Buf-s-14 (pink boxes) and buf-s-18 (teal boxes) are basically indistinguishable. n=3 biological replicates (donors). **b, c** Based on the TOP20 expressed genes of the solvent control (**b**), the expression profile of HCF heavily depends on genes involved with adhesion, migration and ECM function (**c**). Mean ± 95% CI; red boxes indicate membership of the gene in the respective pathway. **d** Same data as in Fig. 5g, but graph shows absolute expression as well as treatment with buf-s-14 and buf-s-18. In contrast to lyco-s, treatment with buf-s-14 and buf-s-18 is unable to induce repression of most collagen isoforms. Mean ± 95% CI; significance in Two-way ANOVA (p<0.05), adjusted following Dunnett, indicated by asterisk (*, lyco-s), single dagger (†, buf-s-14) or double dagger (‡, buf-s-18).
